# Supplementary material for: Noma (cancrum oris): A scoping literature review of a neglected disease (1843 to 2021)
Source: PLoS Negl Trop Dis. 2021 Dec 14;15(12):e0009844. doi: 10.1371/journal.pntd.0009844 (PMC8670680; doi:10.1371/journal.pntd.0009844)
Supplement: S1 Data — (DOCX) [file pntd.0009844.s002.docx]

**S1 Data: Full list of included articles**

| **Title** | **Year of publication** | **Geographic location of study** |
| --- | --- | --- |
| Remarks on cancrum oris and the gangrenous erosion of the cheek of Mr. Dease and Dr. Underwood, and more particularly on the efficacy of the chlorate of potash in the treatment of those diseases | 1843 | United Kingdom |
| Case of cancrum oris: with necrosis of a large portion of the inferior maxillary bone, followed by recovery | 1852 | United Kingdom |
| On the administration of Mercury in cases of cancrum oris | 1852 | United Kingdom |
| Cancrum oris 1856 | 1856 | Not clear |
| On cancrum oris | 1862 | United Kingdom |
| Royal Hospital for sick children, Edinburgh. case of cancrum oris; recovery | 1868 | United Kingdom |
| Observations on the inflammations of the mouth in children, with an illustrative case of cancrum oris. | 1872 | United Kingdom |
| A case of broncho-pneumonia,: followed by cancrum oris and subsequent necrosis of jaw ; recovery | 1872 | United Kingdom |
| Cancrum oris in an European soldier; enlarged spleen; pancreatic disease post-mortem results | 1882 | India |
| Cancrum oris, and its successful treatment by the local application of corrosive sublimate | 1889 | United Kingdom |
| A case of acute malarial poisoning, enteric fever ensuing. complicated by brain and lung symptoms, also by extensive cancrum oris. | 1891 | India |
| Cancrum oris 1891 | 1891 | United Kingdom |
| A case of cancrum oris, with a short critique on the disease | 1893 | Scotland |
| Cancrum oris, followed by extensive ulceration of the cheek and ankylosis of the jaw: recovery | 1893 | United Kingdom |
| Case of cancrum oris | 1893 | United Kingdom |
| A case of cancrum oris in Typhoid fever | 1894 | United Kingdom |
| Locomotor ataxia with cancrum oris as a fatal complication | 1899 | United Kingdom |
| Cancrum oris successfully treated by excision of the cautery | 1901 | United Kingdom |
| Cancrum oris with its common complications | 1901 | United Kingdom |
| A case of cancrum oris affecting both sides | 1902 | United Kingdom |
| Cancrum oris | 1907 | United States of America |
| Noma (cancrum oris) in an adult, with report of a case | 1911 | United States of America |
| Cancrum oris treated by excision and subsequent tube grafting | 1923 | Myanmar |
| Cancrum oris in a case of leukæmia | 1926 | Nigeria |
| A case of stiff jaw after cancrum oris-surgical interference-cure | 1927 | India |
| A case of cancrum oris following typhoid fever; with plastic repair | 1931 | Canada |
| A case of cancrum oris | 1931 | United Kingdom |
| An unusual case of cancrum oris | 1935 | India |
| A case of noma (cancrum oris) complicating non-specific ulcerative colitis | 1935 | United States of America |
| A case of cancrum oris as a complication of bacillary dysentery | 1937 | India |
| A case note on cancrum oris following pneumonia treated by prontosil | 1938 | India |
| Penicillin in cancrum oris complicating kala-azar | 1945 | India |
| A case of cancrum oris; survival after treatment with penicillin | 1947 | South Africa |
| Use of penicillin in cancrum oris | 1947 | Bangladesh |
| Cancrum oris | 1949 | One case in Iraq, one in India |
| Cancrum oris among African natives | 1949 | Zimbabwe |
| Oral aureomycin in the treatment of tropical ulcers and cancrum oris | 1950 | Ghana |
| Infective gangrene of the mouth (cancrum oris) | 1952 | Nigeria |
| Noma or gangrenous stomatitis: report of a case | 1956 | United States of America |
| Noma: report of a case | 1961 | United States of America |
| Cancrum oris. a clinical survey of 20 cases | 1966 | India |
| Cancrum oris-like lesion associated with acute myelogenous leukemia | 1974 | United States of America |
| Dermatology in Shiraz, Iran | 1975 | Iran |
| Nomalike lesion in a patient with chronic lymphocytic leukemia: review of the literature and report of a case | 1976 | United States of America |
| Bypassing a problem airway | 1983 | Nigeria |
| Noma: report of two cases | 1983 | Micronesia |
| An interesting case of adult facial gangrene (from papua, new guinea). | 1985 | Papua, New Guinea |
| Noma in children with severe combined immunodeficiency | 1986 | United States of America |
| Sketches of surgical cases drawn in 1884-87 at the east london hospital for children, shadwell. | 1986 | United Kingdom |
| Cancrum oris in an adult caucasian female | 1989 | United Kingdom |
| Necrotizing ulcerative gingivitis and cancrum oris (noma) in Ibadan, Nigeria | 1990 | Nigeria |
| Residual facial deformity resulting from cancrum oris: a case report. | 1994 | Kenya |
| Cancrum oris in an HIV-positive patient | 1995 | United States of America |
| Noma (cancrum oris) in Human Immunodeficiency Virus/acquired immune deficiency syndrome patients: report of eight cases | 1996 | Zimbabwe |
| Cancrum oris-a 35-year retrospective study | 1997 | South Africa |
| Cancrum oris: management, incidence, and implications of human immunodeficiency virus in Zambia | 1998 | Zambia |
| Noma: the Sokoto approach | 1998 | Nigeria |
| Recurrent parotid abscess formation 8 years after an episode of cancrum oris | 1998 | India |
| Total loss of upper and lower lips: challenges in reconstruction | 1998 | Zambia |
| A survey of cases of cancrum oris seen in Ile-Ife, Nigeria | 1999 | Nigeria |
| Awake tracheal intubation through the intubating laryngeal mask | 1999 | Japan |
| Isolation of fusobacterium necrophorum from cancrum oris (noma) | 1999 | Nigeria |
| Microbiological understandings and mysteries of noma (cancrum oris) | 1999 | Nigeria |
| Noma: public health problem in Senegal and epidemilogical surveillance | 1999 | Senegal |
| Pathogenesis of cancrum oris (noma): confounding interactions of malnutrition with infection | 1999 | Nigeria |
| Radial forearm fasciocutaneous free flap as a solution in case of noma | 1999 | Ivory Coast |
| Combination intraoral and extraoral prosthesis used for rehabilitation of a patient treated for cancrum oris: a clinical report | 2000 | South Africa |
| The prefabricated superficial temporal fascia flap in noma surgery | 2000 | Nigeria |
| Noma (cancrum oris): case report in a 4-year-old hiv-positive South African child | 2000 | South Africa |
| Cancrum oris in a caucasian male with type 2 diabetes mellitus | 2001 | Wales |
| Cancrum oris in HIV infected children in Lesotho: report of two cases | 2002 | Lesotho |
| Cancrum oris: its incidence and treatment in Enugu, Nigeria | 2002 | Nigeria |
| An estimation of the incidence of noma in North-West Nigeria | 2003 | Nigeria |
| HIV/AIDS orofacial lesions in 156 Zimbabwean patients at referral oral and maxillofacial surgical clinics | 2003 | Zimbabwe |
| New split scar cheek flap in reconstruction of noma sequelae | 2003 | Niger |
| Noma (cancrum oris) associated with oral myiasis in an adult | 2003 | Brazil |
| Noma: experiences with a microvascular approach under west african conditions | 2003 | Nigeria |
| Reviewing trends in the incidence of cancrum oris in Ibadan, Nigeria | 2003 | Nigeria |
| Cancrum oris and acute necrotising gingivitis complicating HIV infection in children | 2003 | Nigeria |
| Cancrum oris (noma) in a malnourished HIV-positive child from rural KwaZulu-Natal | 2004 | South Africa |
| Cancrum oris | 2004 | India |
| Report of an infant with noma (cancrum oris) | 2004 | Turkey |
| Noma: life cycle of a devastating sore - case report and literature review | 2005 | India |
| Pseudomonas sepsis with noma: an association | 2005 | India |
| Submental intubation for cancrum oris: a case report | 2005 | India |
| Temporal relationship between the occurrence of fresh noma and the timing of linear growth retardation in nigerian children | 2005 | Nigeria |
| Pro- versus anti-inflammatory cytokine profile in african children with acute oro-facial noma (cancrum oris, noma) | 2005 | Nigeria |
| Cancrum oris: a case report | 2006 | India |
| The use of the pedicled supraclavicular flap in noma reconstructive surgery | 2006 | Nigeria |
| Noma (cancrum oris) in Human Immunodeficiency Virus infection and acquired immunodeficiency syndrome (HIV and AIDS): clinical experience in Zimbabwe | 2008 | Zimbabwe |
| Noma in Laos: stigma of severe poverty in rural Asia | 2008 | Laos |
| Recurrent noma (cancrum oris) in Human Immunodeficiency Virus infection and acquired immunodeficiency syndrome (HIV and AIDS): report of a case | 2008 | Zimbabwe |
| Cancrum oris in developed countries | 2009 | Italy |
| Spanish: Mandibular ankylosis: a noma frequent sequel | 2009 | Equatorial Guinea |
| Protocol for managing acute cancrum oris in children: an experience in five cases | 2009 | Nigeria |
| Cancrum oris in an adult with Human Immunodeficiency Virus infection: case report | 2010 | Kenya |
| Case report journey of a noma face | 2010 | Laos |
| Long-term results of trismus release in noma patients | 2010 | Nigeria |
| A 40-year-old man with a perforated cheek | 2011 | United States of America |
| Cancrum oris in a boy with down syndrome | 2011 | Italy |
| Facial reconstruction in the developing world: a complicated matter | 2011 | Ethiopia |
| Gillies fan flap for the reconstruction of an upper lip defect caused by noma: case presentation | 2012 | Nigeria |
| Noma in an Afghani child: a case report | 2012 | Afghanistan |
| HIV and noma in Burkina Faso | 2012 | Burkina Faso |
| Microarray analysis of microbiota of gingival lesions in noma patients | 2013 | Niger |
| Noma (cancrum oris): a report of a case in a young AIDS patient with a review of the pathogenesis | 2013 | South Africa |
| Risk factors for noma disease: a 6-year, prospective, matched case-control study in Niger | 2013 | Niger |
| Noma affected children from Niger have distinct oral microbial communities based on high-throughput sequencing of 16s RRNA gene fragments | 2014 | Niger |
| French-[Noma and Burkitt disease; a particular association about three observations seen in the teaching hospital center Yalgado Ouedraogo (Burkina Faso)] | 2014 | Burkina Faso |
| French-[Evoluting form of cancrum oris, about 55 cases collected at the academic hospital Yalgado Ouedraogo of Ouagadougou] | 2014 | Burkina Faso |
| Reconstruction of complex oro-facial defects using the myocutaneous sub-mental artery flap | 2014 | East Africa |
| Noma: a disease of poverty presenting at an urban hospital in the United States | 2015 | United States of America |
| French- Rehabilitation of noma sequelae | 2015 | Burkina Faso |
| Cancrum oris (noma) in an HIV positive adult: a case report and literature review | 2016 | South Africa |
| Cancrum oris (noma): the role of nutrition in management | 2016 | Cameroon |
| Management of noma: practice competence and knowledge among healthcare workers in a rural district of Zambia | 2017 | Zambia |
| Noma in an HIV infected patient in Guinea-Bissau: a case report | 2017 | Guinea-Bissau |
| Risk factors and mortality rate of acute cancrum oris (noma) in Sokoto north‑west nigeria: a 13‑year survey | 2017 | Nigeria |
| Pattern of tissue destruction among patients diagnosed with cancrum oris (noma) at a northwestern Nigerian hospital, Sokoto | 2017 | Nigeria |
| Cancrum oris (noma): an early sign of acute lymphoblastic leukemia relapse | 2018 | India |
| Noma surgery | 2018 | Nigeria |
| The surgical management of extra-articular ankylosis in noma patients | 2018 | Niger and Burkina Faso |
| An unusual case of noma caused by Klebsiella Pnuemoniae and its management | 2018 | India |
| A progressive ulcer in immunocompetent man: cancrum oris | 2018 | India |
| Risk factors for diagnosed noma in northwest Nigeria: a case-control study, 2017 | 2018 | Nigeria |
| Pattern of noma (cancrum oris) and its risk factors in northwestern Nigeria: a hospital-based retrospective study | 2019 | Nigeria |
| ‘I treat it but i don’t know what this disease is’: A qualitative study on noma (cancrum oris) and traditional healing in northwest Nigeria | 2019 | Nigeria |
| Atypical orofacial necrosis of unknown aetiology: a case report with features similar to noma | 2019 | Scotland |
| Chronic lymphocytic leukemia revealed by a rare complication: noma. First description from Togo | 2019 | Togo |
| Estimated incidence and prevalence of noma in north central Nigeria, 2010–2018: a retrospective study | 2019 | Nigeria |
| Noma in a boy with septic shock: a case report | 2019 | China |
| Noma—knowledge and practice competence among primary healthcare workers: a cross-sectional study in Burkina Faso | 2019 | Burkina Faso |
| The prevalence of noma in northwest Nigeria | 2019 | Nigeria |
| Release of extra articular ankylosis of jaws as a sequelae of cancrum oris with extensive gingival myasis in a scoliosis patient: a rare case report | 2019 | India |
| Rehabilitation of severe maxillary noma defect with zygomatic implants: a case report | 2019 | Vietnam |
| Sociodemographic characteristics of traditional healers and their knowledge of noma: a descriptive survey in three regions of Mali | 2019 | Mali |
| Pattern of noma (cancrum oris) and its risk factors in northwestern Nigeria: a hospital-based retrospective study | 2019 | Nigeria |
| Airway management through a facial defect resulting from noma (orofacial gangrene): a case report | 2020 | Burundi |
| Anaesthetic care for noma (cancrum oris) - the disease, the airway and how to provide anaesthetic care without a clinical safety infrastructure | 2020 | Nigeria |
| Case report: malignant transformation of noma: repair by forearm flap | 2020 | China |
| Distraction therapy to correct trismus following noma | 2020 | Niger |
| Facing Africa: Describing noma in Ethiopia | 2020 | Ethiopia |
| Language and beliefs in relation to noma: a qualitative study, northwest Nigeria | 2020 | Nigeria |
| Outcomes at 18 mo of 37 noma (cancrum oris) cases surgically treated at the Noma Children’s Hospital, Sokoto, Nigeria | 2020 | Nigeria |
| Case report: a rare case of noma (cancrum oris) in a Malian woman | 2021 | Mali |
| Columella reconstruction using double nasolabial flap and costal cartilage: a case report | 2021 | Indonesia |
| Model of care, Noma Children's Hospital, Sokoto, Nigeria | 2021 | Nigeria |
