# Supplementary figures and images for: Noma (cancrum oris): A scoping literature review of a neglected disease (1843 to 2021)
Source: PLoS Negl Trop Dis. 2021 Dec 14;15(12):e0009844. doi: 10.1371/journal.pntd.0009844 (PMC8670680; doi:10.1371/journal.pntd.0009844)

S1 Equation: WHO formula


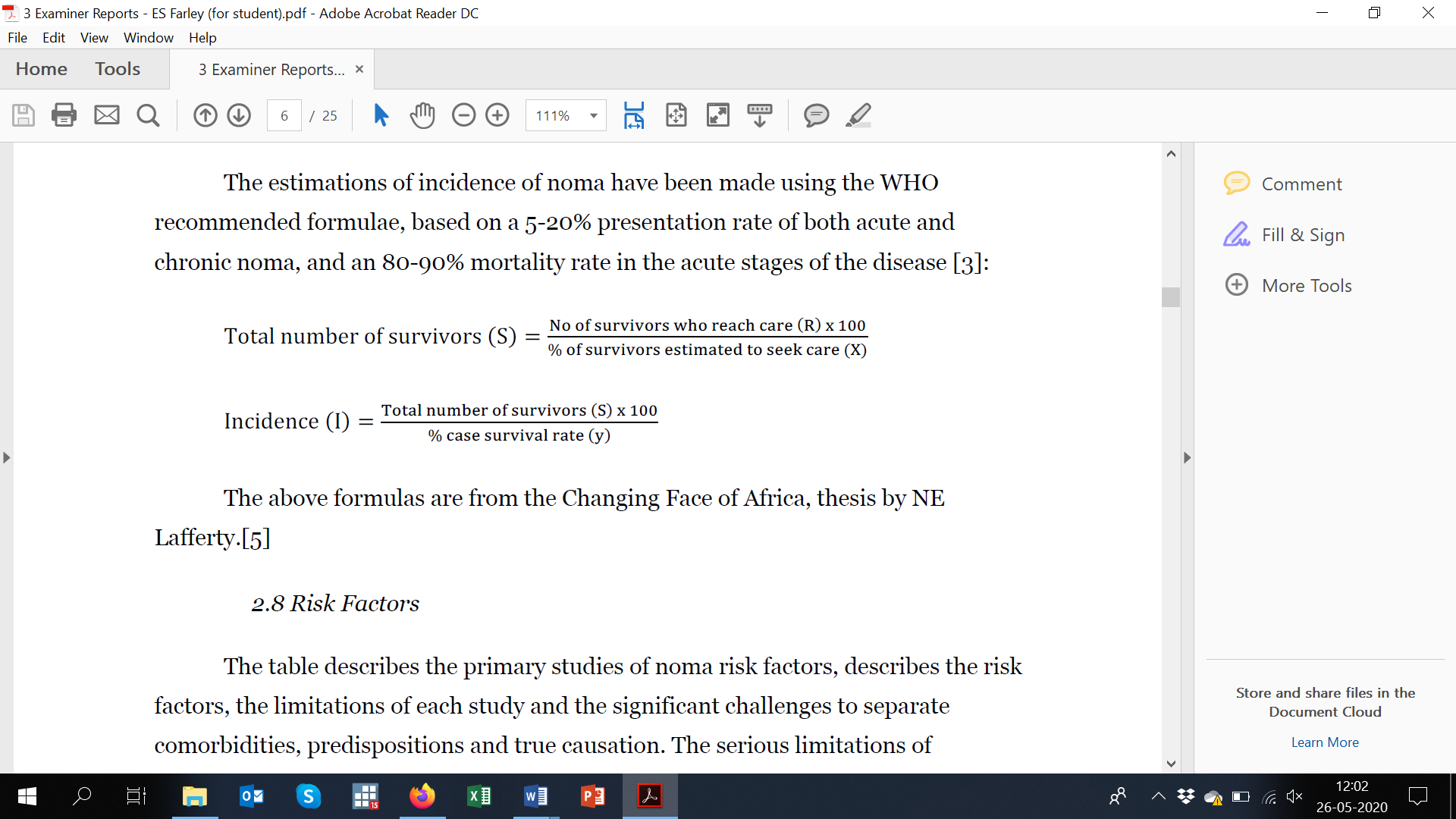

Supplement: S1 Equation — (DOCX) [file pntd.0009844.s003.docx]
