# Supplementary material for: Noma (cancrum oris): A scoping literature review of a neglected disease (1843 to 2021)
Source: PLoS Negl Trop Dis. 2021 Dec 14;15(12):e0009844. doi: 10.1371/journal.pntd.0009844 (PMC8670680; doi:10.1371/journal.pntd.0009844)
Supplement: Attachment — Submitted filename: PLOS NTD response letter.docx [file pntd.0009844.s004.docx]

Elise Farley

Médecins Sans Frontières, Nigeria

Email: noma-research@oca.msf.org

Phone: +27 79 5601 251

To: The Editor-in-Chief

Dear Dr. Joseph M. Vinetz,

# I am writing in relation to the attached manuscript ‘Noma (cancrum oris): a scoping literature review of a neglected disease (1843 to 2021)’ for your consideration for publication in the PLOS NTD

Thank you so much for your review of our manuscript. The reviewers comments are helpful and will make the manuscript stronger.

Please find the following documents enclosed in our submission:

1) Responses to all reviewer comments (below)
2) Tracked changes version of the manuscript
3) Clean version of the manuscript

When we had originally submitted the manuscript, we had applied for a fee waiver. We were wondering if we had been granted a waiver for this manuscript?

We hope that you will continue to consider our request for publication favourably and look forward to your reply.

Sincerely,

On behalf of the author group,

Elise Farley (corresponding author)

Epidemiologist, Médecins Sans Frontières, Nigeria

**Reviewer comments in Green**

**#Author responses in black**

<b>Key Review Criteria Required for Acceptance?</b></br></br> As you describe the new analyses required for acceptance, please consider the following:</br></br>

<b>Methods</b></br></br>

-Are the objectives of the study clearly articulated with a clear testable hypothesis stated?</br> -Is the study design appropriate to address the stated objectives?</br> -Is the population clearly described and appropriate for the hypothesis being tested?</br> -Is the sample size sufficient to ensure adequate power to address the hypothesis being tested?</br> -Were correct statistical analysis used to support conclusions?</br> -Are there concerns about ethical or regulatory requirements being met?</br></br>

Reviewer #1: Yes. The review strategy is clear

Reviewer #2: (No Response)

**#** Author response: Thank you for your review of our article.

--------------------

<b>Results</b></br></br>

-Does the analysis presented match the analysis plan?</br> -Are the results clearly and completely presented?</br> -Are the figures (Tables, Images) of sufficient quality for clarity?</br></br>

Reviewer #1: In the earlier reports ( lines 161-164) there is a hint that this was seen in adults formerly e.g. in soldiers . Is this correct ?

#Author response: Thank you for pointing this out. Noma mostly affect children aged between two and five years, however, cases in immunocompromised adults do occur. We have made several changes in the manuscript to make sure this is clear.

The main change is in the Background section of the manuscript which now reads: “Noma mostly affects disenfranchised children who lack access to basic nutrition, hygiene services and health care, although cases are reported in immunocompromised adults.”

The other changes are all minor (and have been made in tracked changes).

Line 204 Is it possible to be more precise about the definitions of the earliest signs eg gingivitis and ulceration. How extensive is this before there is oedema of the facial skin or do we know this ?

#Author response: Thanks for this point. We have included explanations for the WHO stages in the Clinical Progression section of the manuscript, and readers can also refer to the WHO booklet for further information. Unfortunately the progression of noma, especially its early stages, are really difficult to study and there is a limited amount of research on this.

Activity lines 258-271. In the more recent oral microbiome studies did any analyse the bacterial flora species level, as in other situations, variation in species levels within a single genus can be significant?

#Author response: The microbiology of noma is one of the largest gaps in knowledge around the disease. There are very few studies, and all those we found are detailed in the review. This is one area that needs urgent attention by the research community.

347-350. Can you enlarge on this section of stigma. For instance provide more information on societal attitudes including discrimination against patients.

#Author response: Thank you for this important point. We have now included more information on the discrimination noma patients and their families face as follows:

*“The aesthetic and functional sequelae of noma are compounded by the psychological impacts of the disease, not only on the patient, but also on family members and caretakers. Studies have reported that noma has led to mental health issues due to the social isolation and shunning of survivors and their families, bullying, a lack of access to education, difficulties finding jobs, and limited marital prospects [16, 44, 77, 93–98]. “*

Reviewer #2: (No Response)

--------------------

<b>Conclusions</b></br></br>

-Are the conclusions supported by the data presented?</br> -Are the limitations of analysis clearly described?</br> -Do the authors discuss how these data can be helpful to advance our understanding of the topic under study?</br> -Is public health relevance addressed?</br></br>

Reviewer #1: For successful intervention it would be important either to devise a preventive strategy or devise a system for recognising children whose oral condition is at risk of progression. Do mother and child services have a role to play?

#Author response: This is true and we think that intentions using existing mother and child services would be beneficial.

Is it possible to provide a brief list in table form showing items that could be addressed in order to make a difference?

#Author response: Thank you for this suggestion but we feel we have laid out the areas that need to be addressed in the Discussion in the following paragraph:

*“Based on this literature review, some of the main gaps in knowledge are enumerating the burden of disease (both incidence and prevalence); describing the true mortality rate and pathogenic cause(s) of noma and the role of different comorbidities (specifically measles and HIV) and social structures inplay the development of noma, a finding similar to other reviews [2, 13, 42, 82, 108, 129–131]. Factors that influence prognosis and the long-term outcomes after care (surgical and non-surgical) [132] including the most effective antibiotic treatment protocols [91] need to be assessed. The knowledge of health care workers (biomedical and traditional) about noma in high risk areas, the number of medical school and tropical medicine curriculums that include noma; and the role the varying healthcare actors could play in prevention [133] need to be explored. An additional area for future studies would be to compare prevention methods and messaging [38] to identify the most effective mechanisms. Sincere efforts and political will to eliminate extreme poverty could lead to the elimination of this preventable childhood disease.”*

Reviewer #2: (No Response)

--------------------

<b>Editorial and Data Presentation Modifications?</b></br><br/> Use this section for editorial suggestions as well as relatively minor modifications of existing data that would enhance clarity. If the only modifications needed are minor and/or editorial, you may wish to recommend “Minor Revision” or “Accept”.

Reviewer #1: NO

Reviewer #2: In the 1st sentence of the abstract, would place a comma after the word ‘disease’ and before ‘afflicting’

#Author response: We have made this change.

Sentence 2 in the abstract states “in these settings” although those settings have yet to be defined – would suggest rewording to “and this care is often difficult to access, as most cases of noma occur in resource-limited settings.”

#Author response: We have made this change.

In the abstract, I would reverse the order and percentages (placing 1900’s before 1800s) – as currently it is 2000s, then 1800s, then 1900s

#Author response: We have made this change.

In abstract would change aetiology to etiology

#Author response: Thank you for this suggestion but we would prefer to use the spelling aetiology uniformly.

I would reword this sentence in the abstract, as I would not consider these ‘themes’;

Perhaps: These manuscripts often focused on a number of characteristics regarding noma, including: the history and epidemiology of the disease, ….

#Author response: Thank you for this suggestion, we have changed the word ‘themes’ to ‘areas of interest’ as suggested later in the comments section.

In the last paragraph of the abstract I would remove “many” from “many hundreds of years” and simply state “hundreds of years”. Alternatively, you could also (given this is an encompassing review) be more specific and state when it was first reported in the literature

#Author response: We have made this change.

While mentioning this point – I would suggest removing the word “scoping” from the title of this article

\# Author response: Thank you for this suggestion, however we would prefer to leave the word ‘scoping’ in as we want to make it clear that this is a scoping review. We also want to follow the PRISMA guidelines for a scoping literature review and one of the requirements is having the word in the title.

In the article, insert a comma after disfiguring in line 20

#Author response: We have made this change.

Line 22 – in the body, again would comment: “in these settings”; “these settings” have yet to be defined in the manuscript – would suggest rewording to “and this care is often difficult to access, as most cases of noma occur in resource-limited settings.”

#Author response: We have made this change.

Line 22 – would remove the word scoping

\# Author response: Thank you for this suggestion, however we would prefer to leave the word ‘scoping’ in as we want to make it clear that this is a scoping review. We also want to follow the PRISMA guidelines for a scoping literature review and one of the requirements is having the word in the title.

Lines 33-35 – again would change this to be chronological order – so 2000s, then 1900s, then 1800s, rather than 2000s, then 1800s, then 1900s as is currently written

#Author response: We have made this change.

Line 39 – again, would remove “many” from hundreds of years in the literature

#Author response: We have made this change.

Line 47 – would add a comma after the word disfiguring

#Author response: We have made this change.

Line 49- I would remove the 2 words “to sequelae”

#Author response: Thank you for this suggest. We have changed this sentence to: “Once the disease reaches the last stage, sequelae, survivors require expert surgical care…”

Line 53 – remove “many”

#Author response: We have made this change.

Line 58 – place a comma after the word rate

#Author response: We have made this change.

Line 65 – place a comma after services

#Author response: We have made this change.

Line 66 – would remove the word microbiology so the sentence clause reads …poorly understood and debated…

#Author response: Thank you for this suggestion. We have simplified this sentence to read: “The pathogenesis of noma is poorly understood.”

Line 68 – would change disfigurements to disfigurement, and would also place a comma after disfigurement

#Author response: We have made this change.

Line 68- 69 currently written as …..multiple physical impairments such as difficulty in eating, drinking, seeing.

Would change to….and other sequelae, including difficulty with eating and drinking, as well as ophthalmologic and other other visual changes, and difficulty with breathing as a result of upper airway sequelae.

#Author response: Thanks for this suggestion, we have rephrased this sentence as suggested and it now reads: “Once noma progresses past these stages, the sequelae of noma are numerous and include difficulty in eating, drinking, seeing and breathing[1, 2].”

Line 69 – would replace disfigurements with sequelae

#Author response: We have made this change.

Lines 70-71

Currently written: This impose great difficulties on both the survivor and their families. Would suggest rewording to:

Therefore, noma is associated with a high degree of morbidity for survivors, and this often has a significant impact on family members given the long-lasting and often permanent sequelae.

#Author response: We have made this change.

Line 73 – would remove the word scoping

\# Author response: Thank you for this suggestion, however we would prefer to leave the word ‘scoping’ in as we want to make it clear that this is a scoping review. We also want to follow the PRISMA guidelines for a scoping literature review and one of the requirements is having the word in the title.

Line 107 – suggest replacing themes of interest with areas of interest

#Author response: We have changed the word ‘themes’ to ‘areas of interest’ throughout the manuscript.

Line 114 – would carry through and suggest placing “themes” of interests with areas

#Author response: We have changed the word ‘themes’ to ‘areas of interest’ throughout the manuscript.

Line 115 – aetiology would change to etiology

#Author response: We would prefer to keep the South African and UK spelling of aetiology.

Line 123, insert a comma after 1843

#Author response: We have made this change.

Lines 121-123 – again would reorder so in chronological order as mentioned above

#Author response: We have made this change.

Line 130 129. The word ‘noma’ is derived from a Greek word which, loosely translated, means ‘to devour’

Suggest rewording to : The word ‘noma’ is derived from Greek, and loosely translates to ‘to devour.’

#Author response: Thank you for the suggestion but we would prefer to retain the original sentence.

Line 135

lesion on the skin or an internal mucous surface of the mouth would reword phrase to (lesions on the skin or on the internal mucosal surface of the mouth)

#Author response: We have made this change.

Line 151

Change generalised to generalized

#Author response: We would prefer to use the South African/ UK spelling.

Line 151 – change which to and

#Author response: We have made this change.

Line 152 – replace calls with names

#Author response: We have made this change.

Line 162 – insert a comma after 1900’s

#Author response: We have made this change.

160. Line 164 – change to and in the general population - that is inserting an in in the phrase

#Author response: We have made this change.

Line 168 – insert a comma after the word world

#Author response: We have made this change.

Line 212

Suggest rewording include being aged between two and five years

To being between two and five years of age

#Author response: We have made this change.

Line 213 – suggest adding a hyphen after the word healthcare

#Author response: We have made this change.

Line 215- suggest removing the words of the family from the phrase

#Author response: We have made this change.

Line 215-217, would suggest adding commas, before all the “ands” in these sentences

#Author response: We have made this change.

Line 222, insert comma after variables

#Author response: We have made this change.

Line 231 – changed listed to list

#Author response: We have made this change.

Line 232 change were to are

#Author response: We have made this change.

Line 232 change spelling of anemia

#Author response: We would prefer to keep the South African/ UK spelling.

Line 233-234

This formation slows down the circulatory flow to the gums due to compression, leaving the oral cavity more susceptible to infections [54].

#Author response: I was not sure what change was suggested in this sentence and so I left it as is.

Line 245 – you reference the weaning period – the weaning period from breastfeeding – please define if that is the case

#Author response: We have made this change.

Line 248 – diarrheal - spelling

#Author response: We would prefer to keep the South African/ UK spelling.

Line 254 – would finish the sentence… potential risk factors in some studies

#Author response: We have made this change.

Line 258 – would change atiology to etiology

#Author response: We would prefer to keep the South African/ UK spelling.

line 259 - The pathogenesis of noma is poorly understood and the microbiology is debated would change to The pathogenesis of noma is poorly understood and debated

#Author response: We have made this change.

line 264 – place a comma after patients

#Author response: We have made this change.

line 265 – place a comma after consistently line

#Author response: We have made this change.

266-267 currently starts with Other studies have noted that noma incorporates the characteristics of an opportunistic infection, Would suggest rewording this clause to Other have noted that the characteristics of noma are similar to that of an opportunistic infection

#Author response: We have made this change.

Line 269

or immunodeficiency states

rephase to

or occur in immunocompromised individuals

#Author response: We have made this change.

line 269 – what are where are the concurrent infections reference – please comment on this and provide a citation

#Author response: We have made this change.

line 277 change manifestation to manifestations

#Author response: We have made this change.

line 277 change to “are unique”

#Author response: We have made this change.

line 278 starts as an inflammation of the gums change to starts with inflammation of the gums

#Author response: We have made this change.

line 28 - lasting only a couple of weeks would define this further lasting only several weeks or lasting 1-2 weeks or or lasting 3-4 weeks or other…

#Author response: The evidence did not provide a concrete answer on this and so we would prefer to leave the sentence as is.

line 288 – insert a comma after debridement

#Author response: We have made this change.

line 288 insert a comma after early

#Author response: We have made this change.

line 291 – would replace disfigurements with deformities

#Author response: We have changed this to ‘sequelae’.

line 291 – 292 – this is repetitive (the exact same sentence I believe as above) – see my recommendations on change this sentence, and whether it needs to be repeated again

#Author response: I could not see the duplication in this sentence.

line 295 – insert a comma after airway

#Author response: We have made this change.

line 295 – 296 suggest rewording sentence to :

The deformities that result from noma can lead to social stigmatization

#Author response: We have changed this sentence to: “The aesthetic and functional sequelae of noma are compounded by the psychological impacts of the disease, not only on the patient, but also on family members and caretakers. Studies have reported that noma has led to mental health issues due to the social isolation and shunning of survivors and their families, bullying, a lack of access to education, difficulties finding jobs, and limited marital prospects [16, 44, 77, 93–98].”

Line 305 - biomedical I am not sure what biomedical institutions are – can you define this or further define

#Author response: We have now included more information in thie sentence as follows: “(hospitals, clinics)”

Line 307 – insert a comma after leeches

#Author response: We have made this change.

Line 328 defects [102] that need a comprehensive suite of treatments Suggest rewording to Defects that often require a number of surgical treatments

#Author response: We have made this change.

Line 329 – suggesting removing the word provision

#Author response: We have made this change.

Line 367 – I see the use of biomedical again – perhaps I would just change all these biomedical to medical all these implies western medicine, rather than herbal treatments as noted

#Author response: We have made this change.

Line 368…..patients, and themselves attending didactic training sessions on noma.

#Author response: We have made this change.

Line 371 – There is limited evidence around the pathogenesis of noma leading to death Reword to There is limited and inconclusive data regarding the risk factors that are associated with mortality

#Author response: We have made this change.

Line 373 – suggest changing enumerated to understood

#Author response: We would prefer to leave it as enumerated as we are discussing a figure.

Line 376 – suggesting removing about from the sentence

#Author response: We have made this change.

Line 378

Replace timeously with in a timely manner

#Author response: We have made this change.

Line 382 – insert a comma afte (49-94%)

#Author response: We have made this change.

Line 384

chart reviews, no

change to

chart reviews; no

#Author response: We have made this change.

line 386 – insert a comma after mind

#Author response: We have made this change.

line 402 – replace our ability with the ability

#Author response: We have made this change.

line 413

surgical and non-surgical) insert a comma after this phrase

#Author response: We have made this change.

line 414 – insert a comma after protocols

#Author response: We have made this change.

sentence starting 415

suggesting rewording to :

Healthcare curriculums, both for practicing clinicians as well as trainees, should include noma – with salient information on the pathogenesis, known risk factors, and importance of timely diagnosis and treatment.

#Author response: We would prefer to leave the sentence as is, as what we are trying to say is that those areas need to be explored, and while we agree that noma should be added to the curricula, we were wanting to explain needs for future research.

Line 418 – insert a comma after messaging

#Author response: We have made this change.

Line 418 -420

Currently

Sincere efforts and political will to eliminate extreme poverty could lead to the elimination of this preventable childhood.

Suggesting rewording to:

Efforts to eliminate extreme poverty may lead to a reduction in the number of cases of noma, and potentially even eliminate this disease.

#Author response: We have made this change.

Line 422 – replace the review with this review

#Author response: We have made this change.

Line 425 – insert a comma after misinterpretation

#Author response: We have made this change.

Line 429 – add In summary, to start off the first sentence

#Author response: We have made this change.

Line 430, insert a comma after disfiguring

#Author response: We have made this change.

--------------------

<b>Summary and General Comments</b></br></br> Use this section to provide overall comments, discuss strengths/weaknesses of the study, novelty, significance, general execution and scholarship. You may also include additional comments for the author, including concerns about dual publication, research ethics, or publication ethics. If requesting major revision, please articulate the new experiments that are needed.

Reviewer #1: If there is one message that comes out of this thorough review of noma it is that it has truly been neglected.

Reviewer #2: Thank you for this detailed and throughout review of noma. I have included changes that are mostly syntax in nature.

#Author response: Thank you very much for your review and useful suggestions, it is much appreciated.

--------------------

PLOS authors have the option to publish the peer review history of their article (<a href="<https://journals.plos.org/plosntds/s/editorial-and-peer-review-process#loc-peer-review-history>" target="_blank">what does this mean?</a>). If published, this will include your full peer review and any attached files.<br><br>

If you choose “no”, your identity will remain anonymous but your review may still be made public.<br><br>

<b>Do you want your identity to be public for this peer review?</b> For information about this choice, including consent withdrawal, please see our <a href="<https://www.plos.org/privacy-policy>" target="_blank">Privacy Policy</a>.

Reviewer #1: No

Reviewer #2: No
